# Faith, Culture, and Cancer: A Qualitative Study Exploring Muslim Women's Perspectives on Clinical Trials in the United Kingdom

**DOI:** 10.1111/hex.70823

**Published:** 2026-08-18

**Authors:** Lorraine Turner, Sally Taylor, Ashleigh Ward, Nighat Ahmed, Janelle Yorke

**Affiliations:** ^1^ Department of Research & Innovation The Christie NHS Foundation Trust Manchester UK; ^2^ Division of Nursing, Midwifery and Social Work, School of Health Sciences, Faculty of Biology, Medicine and Health The University of Manchester Manchester UK; ^3^ Christie Patient Centred Research The Christie NHS Foundation Trust Manchester UK; ^4^ PPI Independent Contributor Manchester UK; ^5^ School of Nursing The Hong Kong Polytechnic University Hong Kong, SAR China

**Keywords:** cancer, clinical trials, culture, Muslim, qualitative research

## Abstract

**Introduction:**

Despite breast cancer disproportionately affecting ethnically diverse populations, these groups remain underrepresented in UK cancer clinical trials, limiting the validity and generalisability of research findings, including treatment efficacy and safety. Around one‐third of the UK's ethnic minority population is Muslim, yet little is known about Muslim women's experiences of breast cancer and clinical trial participation. This study aims to explore how faith, culture and religious beliefs influence Muslim women's experiences, understanding, perceptions and participation in cancer clinical trials.

**Methods:**

Purposive and snowball sampling were used to recruit a diverse group of Muslim women with breast cancer. Women whose first language was non‐English were included. Semi‐structured interviews were conducted either face to face, online Teams or telephone. All participants completed a demographic questionnaire. Research ambassadors, well versed in the research protocol, supported translation and participants' linguistic needs. An inductive reflexive thematic analysis (RTA) was conducted to generate codes and themes, which were then mapped onto the capability, opportunity and motivation behaviour system COM‐B model.

**Results:**

Fifteen Muslim women and three immediate family members were consented and interviewed. Participants were from a wide geographical area across Greater Manchester and Lancashire in the United Kingdom, representing diverse ethnicities, age and social position. Women with primary and secondary breast cancer participated; the majority were undergoing systemic anticancer therapy, and three had taken part in randomised clinical trials. RTA generated five main themes: ‘What defines who I am,’ ‘perception of cancer,’ ‘perception and experience of clinical trials,’ ‘information & education to support decision making,’ and ‘the role of the healthcare professional (HCP) in supporting trial participation.’ Women's capability to participate was shaped by their knowledge of clinical trials and access to information. Opportunities were influenced by sociocultural factors, including gender roles, family expectations and HCPs' cultural competence, while motivation was shaped by women's identity, religion and trust in HCPs.

**Conclusion:**

This study provides insight into Muslim women's health‐related beliefs and behaviours that influence participation in cancer clinical trials. Findings will help identify areas where action can be taken at intrapersonal, interpersonal and community level to help empower women to participate in cancer clinical trials.

**Patient and Public Contribution:**

Ethnically diverse patients and members from community grassroots organisations informed a culturally sensitive protocol. Research ambassadors contributed across the research cycle, including adapting interview materials, supporting recruitment and participation (particularly for non‐English speakers), and providing cultural and religious insight during analysis and interpretation.

## 1 Introduction

Including ethnic communities in research is critical from scientific, policy and ethical grounds. Currently, there is underrepresentation of ethnic groups in research such as clinical trials and cohort studies across all types of disease in the United Kingdom, Europe and the United States [1, 2]. Underrepresentation is a significant concern as it hinders the validity and generalisability of research findings across different populations. This can lead to a lack of data on how different populations respond to treatments, potentially leading to unequal healthcare outcomes, which perpetuates existing health inequalities and suboptimal medical care for ethnic communities [3, 4].

Breast cancer represents a substantial global health burden, with an estimated 2.3 million women diagnosed and approximately 670,000 deaths reported worldwide in 2022 [5]. It is well reported that breast cancer disproportionately affects certain ethnic groups in terms of incidence and outcomes [6, 7]. Muslim women are frequently diagnosed with breast cancer in later stages and have a higher mortality than non‐Muslim counterparts [8]. Despite the disproportionate adverse health outcomes for ethnic minority patients, breast cancer clinical trials historically show poor ethnic diversity amongst participants [9].

Evidence suggests that barriers to cancer clinical trial participation among ethnically diverse populations operate at clinician, patient and system levels [10]. Clinician‐related barriers include unconscious bias, assumptions about patients' willingness or ability to participate, limited awareness of available trials and lack of protective time and resources [11, 12, 13]. Patient‐level barriers include limited awareness of research [14, 15], mistrust [16] and socio‐cultural influences [17, 18, 19], while system‐level barriers include restrictive eligibility criteria, exclusion of non‐English speakers, complex trial procedures and inaccessible trial locations [11, 20, 21, 22, 23].

Health literacy and research literacy are important determinants of informed clinical trial participation [18]. Limited health and research literacy are associated with poorer understanding of cancer information and lower clinical trial participation among ethnically diverse populations [24, 25, 26]. Language and linguistic barriers, culturally inappropriate communication, and increasingly complex participation information sheets and consent forms may further reduce understanding and impede informed decision‐making [18]. In addition, failure to provide information in culturally relevant and accessible formats may also be perceived as disrespectful to diverse ethnic communities [19].

The influence of religion on cancer clinical trial participation among Muslim women remains unexplored. Although religious beliefs have been reported to both facilitate [27, 28] and discourage engagement with healthcare and research [29, 30], Muslims are not a homogeneous population. Religious beliefs are frequently intertwined with cultural practices and other social determinants of health, making it difficult to distinguish the independent influence of religion on research participation [30, 31].

The evidence surrounding barriers and facilitators to clinical trial participation is derived predominantly from the United States, with limited inclusion of non‐English‐speaking participants, reducing its applicability to the UK healthcare context. Consequently, little is known about how cultural and religious influences shape cancer clinical trial participation among ethnically diverse communities in the United Kingdom, particularly Muslim women with breast cancer.

There is limited research examining the intersectionality and heterogeneity amongst ethnic groups and how faith and cultural norms are related to an individual's health beliefs and how these may affect women's decision to participate in cancer clinical trials [15]. Applying an intersectionality lens allows researchers to go beyond individual risks to explore the ways in which individual identities compound with social determinants of health and institutional services influence breast cancer care and outcomes [32].

Understanding individual beliefs from a personal perspective allows for tailored interventions that address specific needs and concerns. Behaviour change theories are used to understand the influences on health behaviours. The capability, opportunity, motivation behaviour (COM‐B) model is commonly used for analysing health behaviours [33, 34]. It states that a person's behaviour is a function of their physical and psychological capability to engage in the activity concerned, their social and physical opportunity (factors that lie outside the individual that make the behaviour possible or prompt it) and their reflective or automatic motivation (brain processes that energise and direct behaviour, not just goals and conscious decision making) to demonstrate their behaviour [35]. If we understand the nature of behaviour that influences Muslim women's participation in cancer clinical trials, then we can transition this understanding to designing and evaluating behaviour change interventions.

This study seeks to address the noted gaps in the literature by exploring Muslim women's experience of cancer, focusing on how faith, religion and cultural norms impact perceptions, understanding and clinical trial participation.

## 2 Methods

### 2.1 Study Design

This qualitative exploratory study employed semi‐structured interviews to explore perceptions and experiences of cancer and participation in clinical trials amongst Muslim women and their family members. It was set in Greater Manchester (GM), UK, where 29% of residents are from diverse ethnic communities [36], and 22% of the population are Muslim [36].

### 2.2 Patient and Public Involvement and Engagement (PPIE)

Table 1 summarises PPIE contributions throughout the study, including the involvement of two female Muslim research ambassadors (RAs).

**Table 1:** Summary of patient, public involvement and engagement (PPIE).

PPIE group	Contributors (*n*)	Representation	Stage of involvement	Key contributions
Greater Manchester VOCAL/Black & Asian Research Advisory Group (BRAG)	5	Patients and community members (Black, Chinese and Pakistani)	Study design	Informed research priorities, study design, protocol development and refinement of research questions.
NHS Trust PPI group & Equality, Diversity & Inclusion (EDI) leads	6	Patients, family members and community members (White British and Pakistani)	Study design	Reviewed participant materials, recruitment strategies, cultural appropriateness and ethical considerations to improve accessibility and inclusivity.
Cancer charities, grassroots organisations, women's community groups, community champions and VCSE representatives	10+	Patients, carers, family members and community members (Pakistani, Bangladeshi, Indian, Black African, Black African‐Caribbean and white British)	Recruitment	Promoted the study through community networks, supported snowball recruitment and dissemination of participant and RA recruitment materials.
Research ambassadors (RAs)	2	Degree‐educated Muslim women recruited from the local community through the NHS Trust PPIE recruitment process following community advertising and local governance procedures	Recruitment & data collection	Received NIHR PPIE, Trust R&I and study‐specific training.
				Supported culturally and linguistically appropriate recruitment, informed consent, interpretation, translation, adaptation of study materials and participant support, enhancing cultural responsiveness throughout data collection.
RAs	2	As above	Data analysis and interpretation	Contributed cultural and religious insight during coding and theme development, supporting interpretation of culturally specific meanings and enhancing the credibility and trustworthiness of the findings.

*Note:* Overall PPIE contribution: 21+ diverse patients, carers and community representatives. PPIE informed all stages of the study, including study design, recruitment, cultural adaptation, data collection, interpretation and dissemination planning, ensuring the research remained culturally responsive and community centred.

### 2.3 Participants

Purposive and snowball sampling were used to recruit Muslim women with breast cancer, reflecting variation in age, ethnicity, social position, and geography. Eligibility criteria included in Table 2.

**Table 2:** Eligibility criteria.

Women (> 18 years) who self‐identified as Muslim with any type and stage of breast cancer receiving treatment (neoadjuvant, adjuvant and palliative) or on follow‐up, with or without prior clinical trial experience.
Residing in Greater Manchester/Northwest England
Participant's immediate family member/primary carer (male or female) nominated by the participant, with participant consent
Willing and able to provide informed consent (verbal or written)

**Figure 1:**
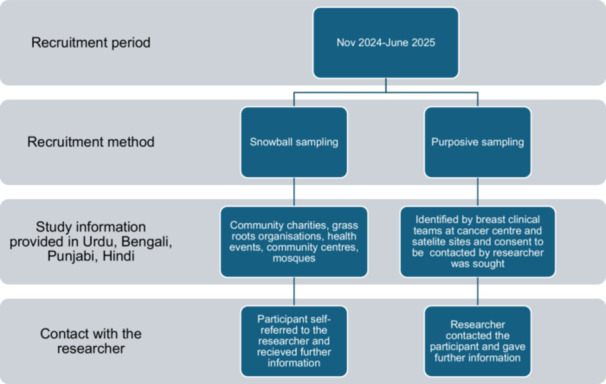
Participant recruitment process.

Recruitment details are provided in Figure 1.

Eligible participants provided verbal and written consent, which included the use of interview recordings consisting of voice only and using an encrypted audio recorder or via the University's approved virtual platform MS 365 Teams. All identifiable information was removed; participants were pseudonymised. Once interviews were transcribed, the audio files were securely deleted. Following consent, all participants completed a demographic questionnaire. The target sample was 12–15 participants, and up to eight family members; 10–15 participants is considered sufficient for qualitative research [37]. The concept of ‘information power’ was used, whereby recruitment was guided by the richness and relevance of the data gathered to answer the study aims [38]. Ethical approval was granted by the South West‐Frenchay Research Ethics Committee, UK (REC Ref 18/SW/0093) and the Health Regulatory Authority & Health and Care Research Wales (Ref: IRAS 343703).

### 2.4 Data Collection

The interview topic guide was based on findings of our previous systematic review [15], refined with PPI input and piloted with two Muslim patients. It covered: (1) introductory questions relating to everyday life to build rapport; (2) cancer experience and family impact; (3) understanding and experiences of clinical trials.

Interviews were conducted in person in participants' homes or in a private room, via Microsoft Teams or telephone. The interviewer (L.T.), known only in her research role, disclosed her background as a Consultant Nurse Practitioner and her motivation to address inequalities in research. She sought to minimise power imbalances by using her first name, dressing informally, removing professional identifiers and spending time with the participants before and after interviews. Having RAs present allowed participants to speak freely in their preferred language. Participation was voluntary, and withdrawal was permitted at any time. L.T. kept a reflexive diary during interviews to draw on during interviews and analysis. Recognising social privilege and acknowledging her positionality as an ‘outsider researcher’ [39], the reflective practice served to acknowledge the researcher's biases and assumptions about the subject matter as a healthcare professional working in breast oncology.

Interviews were audio‐recorded and transcribed verbatim. For Urdu and Bengali interviews, the RA translated while L.T. transcribed. A bilingual researcher independently reviewed translated transcripts. Transcripts were pseudonymised and checked against audio files for accuracy. Notes on emotional tone, pauses and dialect features were added to support interpretation.

### 2.5 Data Analysis

Transcribed interviews were managed using NVIVO (NVIVO 15) and analysed using Braun & Clarke's reflexive thematic analysis (RTA) [40, 41]. An inductive RTA approach was used to generate codes and themes grounded in participants' accounts (Table 3). Formal participant member checking was not undertaken, as this is not considered essential within RTA [42]. Interpretation was strengthened through ongoing reflexive discussions between the research team and RAs. The involvement of RAs facilitated member reflections rather than seeking participant consensus, supporting richer interpretation of participants' meanings [43]. Fully developed themes were deductively mapped to the COM‐B model to provide a theoretically informed interpretation of the behavioural influences on clinical trial participation. This approach is consistent with qualitative health research that applies behavioural frameworks post‐analysis to enhance exploratory depth and support intervention development [33, 44, 45].

**Table 3:** The process of reflexive thematic analysis.

1. Familiarisation	During this phase, the L.T. immersed herself in the dataset, initially transcribing several of the interviews, capturing not only the spoken words but also recorded field notes on non‐verbal cues and the researcher's reflexive notes. The researcher familiarised herself by reading and rereading the interview scripts, noting initial thoughts, ideas and patterns. In addition, recognising and documented biases, assumptions and theoretical positioning.
2. Generating initial codes	Every line of each transcript was systematically coded by L.T. based on semantic similarities. The researcher discussed this inductive coding with S.T. and N.A. Following discussions, codes were renamed and/or combined/split to accurately reflect the nuances of meaning across the codes. NVivo was used to facilitate coding.
3. Generating initial themes	L.T. and S.T. carried out latent analysis by grouping codes together that shared a unifying feature or represented a meaningful pattern and connection in the data to generate initial candidate themes/subthemes. These themes captured a broader, more conceptual level of analysis through an interactive process of examination and interpretation. Candidate themes were discussed with A.W. and N.A. to ensure that the data extracts within each theme were coherent, meaningful and resonated with the experiences of the research team. Thematic maps were used to help visualise the relationship between codes and themes/subthemes. It provided transparency in the researchers' thematic reasoning and aided discussion amongst the wider team.
4. Developing and reviewing themes	L.T. and A.W. reviewed, modified and refined the initial candidate themes, revisiting the original dataset to ensure an accurate summary of concepts reflected the meaning in the data. Some codes were discarded, relocated or merged into a single theme. Themes were organised and reorganised into more prominent (main themes) and subthemes that represent the level of nuance and complexity within the broader themes. Any differences in provisional these/subthemes were discussed, and any ‘unallocated’ or ‘miscellaneous’ codes were put aside to be revisited. Reflection on decisions made during refinement and their alignment with research objectives.
5. Refining, defining and naming themes	L.T., S.T., A.W. and N.A. confirmed the theme names and definitions following a feedback process, with further refinement of the exact meaning of each theme and understanding how it conveys the theme's central concept. Discussion on researcher influence in naming and interpreting themes and on balancing thematic depth with clarity, avoiding vague or overly narrow definitions.
6. Writing up	A bottom‐up, inductive approach was applied where the key themes and subthemes generated by the RTA were then mapped to the Capability, Opportunity, Motivation and Behaviour (COM‐B) Framework [35] to add depth and understanding of why Muslim women may or may not participate in cancer clinical trials. This sequential approach ensured that theme development remained data‐driven, with COM‐B informing interpretation rather than shaping initial analysis. Contextual descriptions of the themes were constructed and reported also using the socio‐ecological framework, existing knowledge, and guided by the exploration of relevant literature. Themes and subthemes were represented with a logical flow to represent a coherent story, embedding participants' responses. Table 5 includes themes and subthemes with examples of participant responses to illustrate the validity of the analysis.

Abbreviations: A.W., research assistant; L.T., main researcher; N.A., research ambassador; S.T., senior research fellow.

## 3 Results

### 3.1 Characteristics of Participants

Fifteen participants were interviewed; 13 chose face‐to‐face. Two had their husbands present, who consented to their data being included in the analysis, and one daughter consented and was interviewed separately. The average length of interviews was 56.25 min (38–82 min).

Demographics are presented in Table 4. Participants were from a wide geographical area across GM and Lancashire, representing diverse ethnicities.

**Table 4:** Demographics of participants and their immediate family members.

Total participants	*N* = 15	Immediate family members (*n* = 3)
Gender	(*n*)	(*n*)
Female	15	1 (daughter)
Male		2 (husbands)
Ethnicity	(*n*)	(*n*)
Indian	1	
Arab	2	1
Pakistani	9	2
Bangladeshi	2	
African	1	
Age groups—Mean age: 49.8 years	(*n*)	(*n*)
18–34 years	2	
35–44 years	3	2
45–54 years	4	1
55–64 years	3	
65–74 years	2	
First language	(%)	(*n*)
English	27%	1
Arabic	13%	
Urdu	40%	2
Punjabi	7%	
Bengali	13%	
Highest level of qualification	(%)	(*n*)
No qualifications	20%	
Secondary school qualifications	13%	
Apprenticeships		
College qualifications	7%	
Diploma/Degree level	46%	
Postgraduate qualification	7%	1
Would prefer not to say	7%	2
Employment status	(%)	(*n*)
Full time (35h+)	13%	2
Part time	20%	
Unemployed	20%	
Self‐employed	7%	
Full‐time parent/carer	7%	
Retired	13%	
Sick leave	20%	
Student		1
Primary carer of a child/children under 18	(*n*)	(*n*)
Yes	8	3
No	7	
Country of birth	(*n*)	(*n*)
Bangladesh	2	
Egypt	1	
England	3	1
Morocco	1	
Nigeria	1	
Pakistan	7	2
Resided in the UK (those born outside the UK) (*n* = 12)	(%)	(*n*)
0–14 years	17%	
15–29 years	50%	2
30+ years	33%	
English indices of deprivation (IMD) 2025 (Gov.UK)^a^	(%)	(*n*)
1–2 (most deprived)	67%	2
3–4	7%	
5–6	7%	
7–8	7%	1
9–10 (least deprived)	13%	
Receiving systemic anticancer therapy (SACT) (*n* = 10)	(*n*)	
Primary breast cancer	6	
Contralateral (primary) breast cancer	1	
Secondary breast cancer	3	
Participated in a randomised controlled clinical trial	(*n*) 3	

a IMD: Represents socioeconomic deprivation, based on participants' postcode (geographical area), not as an individual indicator.

#### 3.1.1 Themes

**Figure 2:**
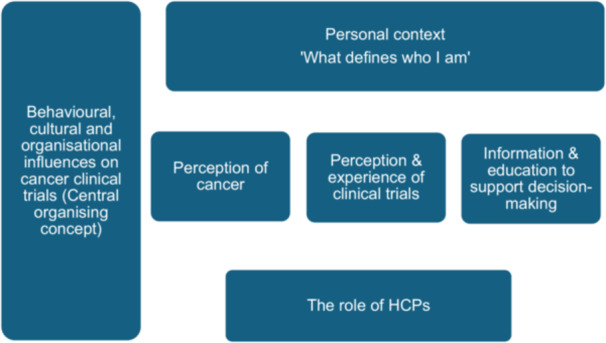
Inductive thematic map of participants' experiences and perceptions of cancer and clinical trials.

Figure 2 presents the key themes and how they interact with the shared central organising concept of how behavioural, cultural and organisational influences affect cancer clinical trial participation. Table 5 provides examples of quotes to support interpretation of each theme/subtheme.

**Table 5:** Sample quotes from semi‐structured interviews.

What defines who I am	Culture	1. ‘Because our community…it's a lot of backbiting, back‐talking…they would spread it to the whole of the community…I didn't want that to happen… You tell somebody something, and they put two, three words extra, and then tell something else…even though their children know about it, but the mum doesn't know about it. Because I know that she is the one who is going to spread it’. (Pt. 14, 48 years, Pakistani, Pakistan) 2. ‘…people [members the community] blow it out of proportion and everyone and his dog would know, they like tell everyone… That's why people… are reluctant about speaking up about it….’ (Pt. 08 Husband, Pakistani, Pakistan) 3. ‘…culturally, really the whole world would have known about it, and everyone would come to your house, I don't want that…I don't want people to ask these questions that I don't want to answer, to be honest’. (Pt. 09, 68 years, Arab, Egypt) 4. ‘Again, and again they phone…they ask from start to finish what the story is. You want to forget but they remind you about the same thing over and over again. A person by remembering then gets upset again… It's hurting ourselves…That's why I shouldn't tell everyone that I have this….’ (Pt. 08, 47 years, Pakistani, Pakistan)
	Role in society and the family	1. ‘I do lots of community work…I work with a deprived community and those women they are… depending on me a lot. They get their strength, their confidence from me. So, it came into my mind, if I share with them that I had cancer…they will think the same thing, oh she will die very soon, so there is no point to come in this organisation, there is no point to rely on her anymore. She is…having bad time, how she will lift us? She doesn't have the power now, she is suffering the killer disease, so how she will pull us, you know, on that way’. (Pt. 06, 49 years, Bangladeshi, Bangladesh) 2. ‘…some of our staff work in the community…I didn't want that pressure… I want to do my business and do it professionally without getting into these emotional things with people. Otherwise, that's it, I can't work. And I can't not work; I want to work’. (Pt. 09, 68 years, Arab, Egypt) 3. ‘…in my house like I'm in charge of…all I could think was oh I need to show them all this stuff and I need to tell them all this stuff…teach them how to cook…I was trying to be practical’. (Pt. 01, 52 years, Indian, England) 4. ‘the first words that came out of my mouth were… “Who's going to look after our mum”…I use to look after her three days a week and she was quite bad with Alzheimer's and dementia…how is she going to cope without me…Because both my brothers worked…I don't even think I thought about myself [participant laughed] I just thought how will they function’. (Pt. 01, 52 years, Indian, England) 5. ‘The worry wasn't about myself but more of who was around me’. (Pt. 02, 59 years, Arab, Morocco) 6. ‘…I wasn't thinking about myself…I was just thinking of the impact on my family…my mind went to my son …the effect it's going to have on him and that it's going to mess his GCSE's up. I think that's what I was worried about more than anything’. (Pt. 11, 45 years, Pakistani, England) 7. ‘…when I had my sternotomy, they [young children] didn't know I was in hospital, they thought I just was away and then became unwell and then ended up in hospital’. (Pt. 03, 43 years, Pakistani, English) 8. ‘Husband is very worried, and my son noticed I'm not as active…he [son aged 6] likes for me to get ready wear nice clothes, put my make up on…Today I have just come like this [attending hospital for bloods prior to treatment], otherwise I'd get ready, and dress up. I put my make up on, take him out and it makes him happy. He says… twirl around as I like it when you do that… and then when I don't feel well…he gets worried as well….’ (Pt. 10, 33 years, Pakistani, Pakistan) 9. ‘…people always think that when someone in the family is diagnosed with cancer, everyone is going to rally around and support them. But when that person is the person that keeps certain practical things of their house going, it doesn't quite work like that….’ (Pt. 01, 52 years, Indian, England) 10. ‘My little two boys didn't know…I was going to tell them before the surgery…in a good way…But I didn't do that, because they were stressing me one day so much…I just broke; I said, do what you want to do, I've got cancer. It really just came out of my mouth…I started crying…It's not a good way, the way I told them….’ (Pt. 14, 48 years, Pakistani, Pakistan) 11. ‘Because the whole family…Our puzzle was broken I would say …and slowly, slowly we try to make it together’. (Pt. 06, 49 years Bangladeshi, Bangladesh) 12. ‘I had…my nieces, they will cook something and then just drop it off…there were always family members…asking, “oh do you want us to do anything for you”… dropping to hospital, picking up your appointments…they were very good’. (Pt. 07, 58, Pakistani, Pakistan) 13. ‘Friends are really good support…with the cooking, cleaning, I don't feel well at time…. looking after/picking up the children from school. Since the medication I have not been able to pick up the children’. (Pt. 05, 39, Pakistani, Pakistan) 14. ‘He understands, he said mummy are you tired? Are you weak? Do you need water? He cares…the kids keep me going, because so many times I'll be like I can't do this, I'm fed up, but my mum will be like, think about your kids, you need to have the treatment’. (Pt. 12, 34 years, Black African, Nigeria) 15. ‘she's done a lot of things for me in our marriage…cooking, cleaning, washing, making breakfast…now it's her time in need, isn't it right that I should do the same for her. If I was to take the chauvinistic mindset, you still have to do everything, even if you can't stand up…you're on your own… that would be a negative and that would be an unhealthy relationship’. (Pt. 08 husband, Pakistani, Pakistan) 16. ‘…if someone came to me and said, actually we've got such a body who can come and do your shopping, or come and do this, then that helps you. But if someone is coming to talk to you for 2 h about this, that and the other, and your stuffs piling up… I just think of the load and the pressure, and it's just making it all worse really. But if someone had come to me and said, actually, there is some charity that does this. They can actually have volunteers that can come and do your shopping, or they come and cook you a meal…You've got then time to do other things like researching or time to be even ill.’ (Pt. 01, 52 years, Indian, England)
	Religion and faith	1. ‘I would often use faith to support my mum…I actually don't know how people do it without it… it's the idea that there's something greater than you that knows more than you, and you just give that…it's almost like a child to a parent… I don't need to bear this responsibility now…you hand over the affairs to God…he's just going to cope with it all because I'm struggling at the moment…it massively helps’. (Fam Pt. 01, 36 years, Arab, England) 2. ‘I turn to faith at every angle; it's a big part of my life anyway and it's just made me stronger’. (Pt. 11, 45 years, Pakistani, England) 3. ‘Because we are Muslim, we believe our God, that is why I leave everything for him…he's the one who will heal and secure us…he'll give us strength and courage, this helps with positive thinking’. (Pt. 08, 47 years, Pakistani, Pakistan) 4. ‘Those tests of life, they're tests of your faith, tests to make you stronger…Allah will never put a burden on you unless he knows that you can manage this burden’. (Pt. 09, 68 years, Arab, Egypt) 5. ‘…in Islam it's very clear that if you're tested and you turn to God, you're loved more…your patience is being tested but you're also being rewarded…so for me it's not such a waste’. (Pt. 03, 43 years, Pakistani, Pakistan) 6. ‘There are things that are mentioned [in the Quran] positive side that we have to make use of the things that have been created for us…like there is science, and obviously that's where it comes the medicine’. (Pt. 02, 59 years, Arab, Morocco) 7. ‘We have a lot of spirituality and a lot of faith and that has as a whole family got us through the last 8 years…We've got to channel any anxiety and fear towards the towards the faith and just pray and pray’. (Pt. 03, 43 years, Pakistani, England) 8. ‘I'm happy to die and she [the nurse in the hospice] said, are you not scared?…The Creator created me, I'm going to Him…I Know no fear’. (Pt. 13, 69 years, Pakistani, Pakistan) 9. ‘I pray and hope…after a few treatments…Dr will ask me, you are fine, you are ok and everything has gone, you are now cancer free…I am just waiting for this statement from the doctors…I cannot deny the Dr has given me a poor prognosis…’. (Pt. 10, 33 years, Pakistani, Pakistan) 10. ‘I'm a Muslim…but so everybody's beliefs and thoughts are different. I don't pray five times daily, but…I pick up my Quran and read. If I'm depressed…It encourages me, it gives me hope… any time I feel down, I just pick up my Quran and pray…so I believe…the enlightenment also, it's not there for some Muslims’. (Pt. 12, 34 years, Black African, Nigeria)
Perception of cancer	Personal experiences	1. ‘My husband's first cousin…she had breast cancer, and she didn't do anything about it. She had multiple lumps, and she could feel them for years… it was end stage…within months…she was gone. So that showed the seriousness of just leaving it’. (Pt. 11, 45 years, Pakistani, Pakistan) 2. ‘Before when people heard the word cancer it was a really scary thing but now there're more treatments and people survive’. (Pt. 15, 44 years, Bangladeshi, Bangladesh)
	Influence of cultural beliefs	1. ‘In our culture [England] if you are having or diagnosed cancer the doctor will directly tell you but in our country [Bangladesh] they don't…here…the patient has a right to know, but in our culture we don't want to…we think he or she will die very soon…families in Bangladesh, even they don't tell the patient…when they know that they have cancer they will die…it's in our culture’. (Pt. 06, 49 years, Bangladeshi, Bangladesh) 2. ‘So, when they [parents] hear the word cancer, they believe you're dying…when I broke the news all they thought was, oh my god, how am I going to afford the medication and the bills? What's the disease, am I dying? …So, they [parents] were really curious, they were scared. People when they hear cancer, they believe you're dying, those are their thoughts, that is their belief’. (Pt. 12, 34 years, Black African, Nigeria) 3. ‘People have tried to hide their illnesses. I know many women, I'm not sure about other faith but I know a Muslim woman they try to keep it to themselves, they don't share this news’. (Pt. 04, 59 years, Pakistani, Pakistan) 4. ‘…this may be a cultural thing, maybe in our culture…. People don't talk about. Oh no, don't mention it… hide it…’. (Pt. 08, Husband, Pakistani, Pakistan) 5. ‘I don't think this is because of the part of body, you know, I think, overall, they feel shame. Because I know if you're going through some other trials in your life, they would keep it secret… They don't want to share with people because they feel other people are going to think low about us, I think’. (Pt. 04,59 years, Pakistani, Pakistan) 6. ‘Even in the older generations, it's not a taboo subject to speak about…It was not because it's taboo, it's just the way that it was respectful for them to not talk about their private parts’. (Pt. 01, 52 years, Indian, England) 7. ‘The hardest part about radiotherapy is undressing yourself every time and laying there topless…it was more the emotional side…as…a Muslim women you're told all your life to cover up, be modest…it's just constant undressing it's like an alien feeling…’. (Pt. 11, 45 years, Pakistani, Pakistan) 8. ‘I was here in the hospital with one of our family friends…they asked me not to share with anybody in the community or anyone because she has her daughter, and her husband has strictly told her not to share with anyone…still no‐one knows that she had the mastectomy, only her family…they know…in our culture…we are not allowed to talk about it, so they don't share’. (Pt. 06, 49 years, Bangladeshi, Bangladesh) 9. ‘I think those that have been born and bred here and educated here, I think. I would be definitely more aware of it, and it would be more easy to talk about it. As soon as I felt the lump, I was showing my husband…I was talking to my sisters…can you feel it too?…Whereas I don't think somebody from the older generation would be saying that to their husbands or somebody from Pakistan that just recently emigrated over, they would be embarrassed about it…’. (Pt. 11, 45 years, Pakistani, England) 10. ‘It's not my fault…why should I be ashamed of…. nothing is my fault… And especially if you're going through this nasty cancer thing…I don't think there should be any shame or shyness…. But I know… women are… there are so many myths and misunderstandings…It's not their fault they were born in that rigid families or circumstances’. (Pt. 04, 59 years, Pakistani, Pakistan) 11. ‘They very disclose about their own bodies even in their own private homes and bedrooms…When some of my friends came to visit me…they saw me wearing…no sleeves…or nothing and my boobs are out here and there…wearing shorts, they nearly had a fit. [We both laugh]. They looked at me and they said, “do you wear that in front of your husband?” I said, excuse me…I think we have children together. They see that as a very different issue…the culture, they see that very different…’. (Pt. 09, 68 years, Arab, Egypt)
Perception and experience of clinical trials	Awareness and understanding	1. ‘Clinical trials is something new to us, not been given any information regarding this. It's not really understood…it's the first we've heard of these words, do not know what clinical trial means’. (Pt. 05, 39 years, Pakistani, Pakistan) 2. ‘…because people like I said, have never heard about clinical trials or…you know they didn't know it existed’. (Pt. 02, 59 years, Arab, Morocco) 3. ‘…The less you know, the more you are scared and worried because you don't know. We're always worried about what we don't know, right. If people that never heard about research…they would straightaway say, oh, they think we're guineapigs, they're going to try on us’. (Pt. 09, 68 years, Arab, Egypt) 4. ‘…just Asians that I can talk about…and my mum or people of that era or even people who are not from the UK as in born and bred here but from abroad…have limited English knowledge and speaking, would not understand what a clinical trial is. I can say that for a fact because even within my family, my extended family, if I was to speak to people who are from Pakistan… they wouldn't know what it is [clinical trial]…Whereas my own family and my brothers…we're all from here and we're educated, we understand what a clinical trial is and…we understand what it involves…’. (Pt. 03, 43 years, Pakistani, England) 5. ‘Different generations, it makes a difference how they perceive the whole thing about getting cancer, clinical trials and stuff like that…younger generations look at It differently’. (Pt. 01, 52 years, Indian, England) 6. ‘I've spoken to some women, and I say, oh, I'm going to have…have chemotherapy, and I'm going to go on this clinical trial; they do have this concept…some people, not all – this thing, “oh, we'll just leave everything to God”…’. (Pt. 01, 52 years, Indian, England) 7. ‘…we've both been to university so we've had more of an education than a lot of people here that might be diagnosed with cancer. And we would still struggle to understand what a clinical trial entails or what it means, what the implications are, what it involves’. (Pt. 03, 43 years, Pakistani, Pakistan) 8. ‘Some people have gone to Uni…got some kind of science background…And some people don't. I could understand all the things that the doctors were saying but my husband, who hasn't gone to Uni, he wouldn't …and I would explain it to him…some haven't even gone to school here. I could imagine how hard it would be’. (Pt. 11, 45 years, Pakistani, Pakistan). 9. ‘Definitely, me and my husband decision. And my sister [participant laughing]…my other sister‐in‐law, she's very vocal because she's a pharmacist…I sent her the information and she goes…the data's not complete yet, it's on the 10th year…so we're only going to see the outcome…and the benefit of this trial after the 10 years. So, we've not got to that point of being sure that…what they're doing is correct or not. So, she was like… you need to go for the chemo… And I said…how are they going to know if people don't sign up for the trial?’ (Pt. 11, 45 years, Pakistani, Pakistan) 10. ‘I think if my mum had asked my dad…my dad would have said, “oh, just leave it, you don't need to do anything else.” And he would have just dismissed it. And I think maybe even my sister or my brother might have just said, “oh mum, you don't need to take anything else, just leave it”…I don't even know if they would have read it or tried to engage in it’. (Fam 01, 36 years, Arab, England) 11. ‘I think some of the strong reasons for our women not encouraged to take part…I would say their husbands or their families would refuse to let them…A lot of them, when they have cancer or have any terminal illness, they suffer, …there's a massive lack of support in the family. They're…left alone to deal with it…It's a culture thing’. (Pt. 09, 68 years, Arab, Egypt) 12. ‘…I understand that drugs have been tested before, and that this medication is the best for her. I feel that you should do the trials’. (Pt. 05, husband, Pakistani, Pakistan) 13. ‘Without research, really, we cannot make improvements in anything. Because you discover things, you use it to benefit human beings at all levels, whatever the research is, and future generations’. (Pt. 09, 68 years, Arab, Egypt) 14. ‘When you say clinical trials, to me it's like experiments…they want to check…how the treatment about cancer is going…it's a positive because to know how to move on and what to do to add more to the treatments…it's a positive one because research gives you more knowledge’. (Pt 12, 34 years, Black African, Nigeria) 15. ‘I am aware…that all the treatments are based not on, you know people from ethnic minorities… all of the research studies are based on…white people or maybe there could be some ethnic minority of that colour…, maybe some studies in America…people of African Caribbean, or Jamaican…are involved in the research there. but most of it…here is not based on the African people or ethnic minorities’. (Pt. 02, 59 years, Arab, Morocco) 16. ‘Even though life and death is in the hands of God…it doesn't make any difference…after they obtain the results from me and apply it to someone else so I'm happy with that. I've got no problem with that because I'm already unwell, and I don't know about my life’. (Pt. 10, 33 years, Pakistani, Pakistan) 17. ‘I always love to enrich myself for helping other people…because definitely this research or clinical trial, anything will help for our future generation so I'm always happy to’. (Pt. 06, 49 years, Bangladeshi, Bangladesh)
	Benefit Vs Risk	1. ‘you're thinking, should I do this, would it be good for me, is it going to hinder me, is it going to knock things back, am I a guinea pig? There's a lot of mistrust’. (Pt. 03, 43 years, Pakistani, Pakistan) 2. ‘If there was a chemotherapy and there was another one and the other one had less side effects then obviously…it would help me with my diagnosis…I would go for it’. (Pt. 07, 58 years, Pakistani, Pakistan) 3. ‘I wanted to try to avoid chemo, really, because I… heard it's not very…it's not easy, you know’. (Pt. 14, 48 years, Pakistani, Pakistan) 4. ‘…if you look at someone who has gone through chemo, it's not very nice…if there was possible treatments that were less damaging to the body, I think a lot of people would consider that. They feel that this treatment is better…there must be something in it for them to believe that’. (Pt. 08, Husband, Pakistani, Pakistan)
	Personal experience of clinical trials	1. ‘…any clinical trials and she [nurse] said “no.” Without opening anything or without giving me the chance that there might be or let me look into it or let's find out… there was just a clear “no”’. (Pt.02, 59 years, Arab, Morocco) 2. ‘I can't just say, oh I had a rubbish experience of a clinical trial…but I would say it's not going to be what you expect…If you think going on a clinical trial they're watching you…if you get side effects…it's not going to be like that’. (Pt. 01, 52 years, Indian, England) 3. ‘I rang…something's not right, on my lungs, and I've got this temperature…they spoke to the Doctor [Principal Investigator]…and I got an email back saying… take a couple of paracetamol…you'll be all right… then next day, something's not right…Because it was Christmas, and this is the impression I got, that it was busy and no one wanted to do it, they say…if you're ill you need to go to A&E…I ended up going to A&E, wasted hours and hours, only for the doctor to say…we can't do the CT scans for you…Don't stay in, because you're going to pick something up here’. (Pt. 01, 52 years, Indian, England)
Information and education to support decision making	Information source	1. ‘I found out from some ladies when I went to the mosque about breast cancer…They explained to me there; I came home and then made an appointment with the GP’. (Pt. 05, 39 years, Pakistani, Pakistan) 2. ‘When I was going through these things…my friends, they started to send me links to go on, you know, the cancer, the helplines…So, I was reading other people's reviews and I was thinking why I'm not thinking like that, because everybody else is complaining, why me, why me, that was a specific thing’. (Pt. 04, 59 years, Pakistani, Pakistan) 3. ‘…I was referred to these…charity, organisation…because when I first got diagnosed, I was given some leaflets, to go and join the group, I felt reluctant joining. So, when a lady called me…and when we talked, I noticed she was also black like me, and I felt relieved. Opening up and talking to them, I felt relieved, I felt happier’. (Pt. 12, 34 years, Black African, Nigeria) 4. ‘I just let whatever the doctors do, and I just let them get on with it…because it's so exhausting’. (Pt.01, 52 years, Indian, England) 5. ‘And I choose not to go and find out information for myself. I just thought that would have been a waste of my time and effort and extra worry, so I did not go to Google like others would’. (Pt. 02, 59 years, Arab, Morocco) 6. ‘I do rely on him [brother] quite heavily, being a medical professional…I think he knew that this was something a bit overwhelming for me [participant getting upset whilst telling her story]…he's my source of information…he provided me with a lot of reassurance…And he explained it a little bit more in detail, so I signed up for it [consented to clinical trial]’. (Pt. 03, 43 years, Pakistani, England) 7. ‘I don't think she would go for any trial…I think she'd find it difficult to understand because she emigrated over here and she relies on her daughter to understand for things, an 18‐year‐old’. (Pt. 11, 45 years, Pakistani, Pakistan) 8. ‘Not everything you send, people read…if they read it, it does not necessarily mean they understood everything that was written. They may misinterpret a word and that's it, then you've lost that person…just sending papers…sometimes people just put it in the bin… don't have time to read it, why should I bother myself?’ (Pt. 09, 68 years, Arab, Egypt) 9. ‘…sending a participant leaflet form…I don't think is well received. It needs to be somebody talking and explaining it in very simple terms, so it doesn't come across scary, and…somebody they already trust… it's not always useful to translate something into another language in written, because you'll have a lot of patients that speak their language, but don't read it…so giving them a leaflet in their native language, doesn't help at all…’. (Fam 01, 36 years, Arab, England) 10. ‘…with your study, it was the consultant that mentioned it…and then it's followed up immediately by yourself…And then they sent out the relevant information and you were really forthcoming if there's any questions and things…I think it's a good model…’. (Pt. 11, 45 years, Pakistani, Pakistan) 11. ‘Information was really good…got leaflets from the Drs, both Drs, Consultant, and breast Consultant speak in our language, explain everything and Muslim they understand what we are saying’. (Pt. 05, Husband, Pakistani, Pakistan) 12. ‘Sometimes the interpreter…used to speak with my mother and father‐in‐law…and the way they say, I would not specifically say the same things the way that they said it…it's down to personal interpretation’. (Pt. 11, 45 years, Pakistani, Pakistan) 13. ‘With a translator…maybe a bit of training on the clinical trial setting, and what it involved and what clinical trials are in first place…a sort of short course…this is what we're going to need you to explain to patients’. (Pt. 03, 43 years, Pakistani, England) 14. ‘…bring them [videos] out to be seen…in GP practice because everyone goes to a GP practice. Or in a chemist, when you are collecting so just say the importance…in a sentence or two. And then a link’. (Pt. 02, 59 years, Arab, Morocco) 15. ‘You can show them some kind of videos, some kind of interesting stories. It could be more attractive for that people, I think’. (Pt. 10, 33 years, Pakistani, Pakistan) 16. ‘…maybe if it's like a small clip, and explain quicker, maybe it's easier for people than reading; because reading, some people don't understand, you know, the words, that you have to look them up…Maybe it's done in a video form…’. (Pt. 14, 48 years, Pakistani, Pakistan)
	Content and clarity	1. ‘I'm conscious that ethnic minority, you know are disadvantaged…you know people do not understand the language, people don't understand English, never mind medical English, which I myself don't understand much’. (Pt. 02, 59 years, Arab, Morocco) 2. ‘…I think the clinical trial side of things, unless it's explained very, very clearly, they might think that oh they're just trying out some new medication on me that might have adverse effects…So, that's the step number one…it needs to be made clear as to what a clinical trial actually is’. (Pt. 03, 43 years, Pakistani, England) 3. ‘…the clinical word…I will say, in our culture if you say anything clinical, they'll think maybe an operation…they will take me for research, they will cut my parts or these things. So, this came into my mind… I would surely say that clinical makes people in our culture a bit, you know, scared’. (Pt. 04, 59 years, Pakistani, Pakistan) 4. ‘…Clinical…in our country…a private clinic, that means the doctors, they do their private treatment, and it's called clinic…I wonder if people associate clinic as more the private and not the actual routine care maybe…’. (Pt 06, 49 years, Bangladeshi, Bangladesh) 5. ‘…I need to know what they are giving me, how's it going to work, what side effects am I going to have, is there effective treatment, what are the chances…’. (Pt 01, 52 years, Indian, England) 6. ‘I don't mind if it has been tested on other women, and also women of my colour, also it's really important. Because, like I said earlier about my tongue, they've not encountered something like this before. So, I would prefer to know how it's reacted on them and the side‐effects…It's important to know’. (Pt 12, 34 years, Black African, Nigeria)
	Delivery	1. ‘I don't know whether you could come up with like a little alumnus of cancer sufferers who then have recovered, and are happy to then build a little community group or something?…and that reassurance that it's someone that looks vaguely like me, maybe not entirely the same, but she understands my background a bit’. (Fam 01, 36 years, Arab, England) 2. ‘Yeah. That would be good [education session in local Mosque]… But ladies don't go a lot to the mosque, you know, you have to arrange it specially…’. (Pt. 14, 48 years, Pakistani, Pakistan) 3. ‘…for me always interaction, face‐to‐face interaction is better. Even if I talk about my community, the women, they also same like me, they will love the workshop, if you do a workshop with a group of women and you can share what's going to be happening…if you keep one interpreter as well’. (Pt 06, 49 years, Bangladeshi, Bangladesh) 4. ‘TV…or radio adverts… some people do listen to that you know… and radio… you are driving you hear so many things… and raise the awareness…that there are clinical trials in every field. And let's not limit it to just cancer patients’. (Pt 02, 59 years, Arab, Morocco) 5. ‘So, there does need to be a lot more information and, yes, starting earlier maybe in, sort of…you know, towards the high school stages with science lessons – research, explaining what it means and the different phases’. (Pt 03, 43 years, Pakistani, England) 6. ‘…in secondary school in Nigeria, there was this club we go, HIV was new…awareness in this every week…we go to different schools, so enlightening other people also in secondary school. So, if the enlightenment is there, going to talk about it more, like a club, even in high schools, so to enlighten about cancer, and not just breast cancer…it's just starting from the roots’. (Pt 12, 34 years, Black African, Nigeria) 7. ‘That reassurance that it's someone that looks vaguely like me, maybe not entirely the same, but she understands my background…so I think it's that exposure to it that helps with being able to understand that person's situation…it needs to maybe just come from people in the community…like a little group of champions’. (Fam 01, 36 years, Arab, England)
**Role of the HCP**	Interpersonal care	1. ‘Lovely people, very friendly. They meet you and greet you with a big smile. And the chemotherapy team, they were marvellous, more than marvellous…I got on so well with them and I got to learn their names…Very friendly, very supportive, very caring, very sensitive to your needs. And I couldn't have got a better way of treatment… Even my consultant…she was great…It was a very positive experience I have to say, very positive’. (Pt. 09, 68 years, Arab, Egypt) 2. ‘…I felt like I'm very blessed…they have a garden, they have plant, flowers and with patient waiting room while I have the radiotherapy…people were easy…drinks, water, squash. So, those make me very happy, oh my God, they are looking after us…and after chemo or after radiotherapy we have to hydrate our body…that's why they are so much caring’. (Pt. 06, 49 years, Bangladeshi, Bangladesh) 3. ‘…the treatment team and… staff are very nice… they care…they are giving me very good treatment…I was very depressed and anxiety…so they are giving me good counselling…everything is very very good…’. (Pt. 10, 33 years, Pakistani, Pakistan) 4. ‘…in our culture…we don't love to go to the doctor…before I diagnosed cancer, I usually did not visit the GP…I haven't had time to go. So, I think it's better when people are having this treatment if they call to the patient and check…I know it's very cost effective maybe, but minimum one month or two months… It may, I think, help’. (Pt. 06, 49 years, Bangladeshi, Bangladesh) 5. ‘Poppy [research nurse] was my consistent…she was there all the time, so she knew what was going on with me’. (Pt. 01, 52 years, Indian, England) 6. ‘…we had a permanent GP…but last year he left. Now it's every time we go it's different doctor…I don't like that…because you have explained everything. And they don't know you,…our previous doctor…they know…I've got the impression they might think I'm making it up…’. (Pt. 14, 48 years, Pakistani, Pakistan) 7. ‘…you don't even see the same consistent doctor; it's like every time a different doctor comes in…the doctor doesn't know anything about me, they don't know where I am, they're just ticking a box… I felt like that quite a lot of times…they were ticking the boxes, whatever they had to do’. (Pt. 01, 52 years, Indian, England)
	Building and maintaining trusting relationships	1. ‘Decision making…for me when I was going through this…I trusted the medical staff…I trusted everybody, and I left to them to do their job…and for me whatever the outcome is reached I accepted it and went along with it… I did not question it’. (Pt. 02, 59 years, Arab, Morocco) 2. ‘Because we, after God…we believe in doctor, I believe in doctor, whatever he is saying is right’. (Pt. 04, 59 years, Pakistani, Pakistan) 3. ‘…a lot of people don't need to make a decision normally, and then you're suddenly giving them something to decide. And they might not have the skills to make that decision, that can be overwhelming for them…’. (Fam 01, 36 years, Arab, England) 4. ‘He [oncologist] explained once you finish your Chemotherapy… your care is going to go back to your consultant [Surgeon] but guess what…. Not a word from the consultant or from the nurse and I've chased them time, and time and time again…. Imagine the worry that I was going through…they just ignored me’. (Pt. 02, 59 years, Arab, North Africa) 5. ‘I was thinking there might be somebody available to come and they will explain to my kids…I was hoping for that; but there wasn't anything…there was a book called…Mum's Lump…they just give me that… I didn't look at it, I didn't read it, I just told them, in a wrong way…’. (Pt. 14, 48 years, Pakistani, Pakistan) 6. ‘…being told twice it's nothing, and then being told suddenly, no, you have it…I wasn't happy about it, because now it was…nearly six months…with nothing, …My mind was, like, it's already spread …it could have been treated earlier… So, it's scary…’. (Pt. 14, 48 years, Pakistani, Pakistan) 7. ‘I feel quite lucky that it hadn't spread…I know that I was…misdiagnosed for several months…the one thing my brother did say at the time was… should you not have done a biopsy when you first saw her rather than giving her antibiotics? And the surgeon did say, yes, you're right, we should've. So, he acknowledged that I had obviously not had that biopsy which I should've had’. (Pt. 03, 43 years, Pakistani, England) 8. ‘I had this feeling but has gone for over a year, had the checks but told it is clear… I had suspicion there… I flagged it up, but I was told it's nothing…I had lost faith in the machine… the symptoms I had in the left breast was similar to the symptoms previously. Nipple inversion…I went back and then I was diagnosed with a cancer in the other breast…It should have been picked up…. I knew there was something wrong…’. (Pt. 05, 39 years, Pakistani, Pakistan) 9. ‘…it is very important that the word trust is there. You have trust and confidence in the system. And that would encourage people to contribute to it. If any of those are broke, I think that's what people…they don't want to participate [in research] because number one, if they…they don't trust, they don't have confidence, that they may risk their health…’. (Pt. 09, 68 years, Arab, Egypt) 10. ‘…I got a temperature one night…my breathing is tight. So, my husband took me to A&E… I had the worst experience of my life… They [A&E nurse] were honestly horrible…I stayed there…for about six, 7 h…I'm type two diabetic…I felt so hungry and needed a drink…I went to one of the nurses…And I said, I'm sorry, I feel dizzy…I really need something to eat…she showed me where the water is and then she took me outside… She said you can go there; there's a machine, you can buy a sandwich if you want. I didn't go because I wasn't well enough to go’. (Pt. 09, 68 years, Arab, Egypt) 11. ‘…I was suspicious at that stage…I thought…this doctor telling me because they want me to join the trial…do Christie's get paid for going on these? Does AstraZeneca pay them to do these trials? Is it in their interest to have as many people going on a trial? That was all… going through my mind…’. (Pt. 01, 52 years, Indian, England)
	Cultural sensitivity and competency	1. ‘they [radiographers] were really sensitive about touching me…they did not touch my breasts at all…And it made a lot of difference. If they were…whipping my breasts around like that, it would have felt really, really hurtful to me… it made a lot [emphasis in her voice] of difference… So, so sensitive in the way they touched you, it was really dignified, thankfully’. (Pt.11, 45 years, Pakistani, England) 2. ‘…the doctor was telling me about chemo…and he said this drug that they're going to use has got alcohol in it. And I said…I'm not sure about this one…is there anything else?…But then they called me in…and they said…they've asked the imam, so fatwa they call it…it is something that you can have…it's not the alcohol you actually drink, it's for medication…And I said, that's fine…’. (Pt. 07, 58 years, Pakistani, Pakistan) 3. ‘…the problem is you can't make any generalisations, even within one community… so all South Asians are not the same, all Arabs are not the same. You go from one house to the next, and it's like you're in a different culture again…’. (Fam 01, 36 years, Arab, England) 4. ‘…we can sometimes forget that everyone is different…that it's really easy when someone comes in front of you and looks a certain way, that we kind of go with that stereotypical, oh, well there's no point mentioning that to her, because she's not going to do it, or there's no point, because they won't have that support. But just to always be really careful about not doing it, and just offer the same to everyone, and let them be the one that says, no, or yes…’. (Fam 01, 36 years, Arab, England) 5. ‘You are always going to naturally feel more…in touch or comfortable, with someone who looks at things…the same as you, that's completely normal. It's just about recognising that that doesn't mean everybody else should be ignored…we just treat everybody the same, but appreciate that I will always, naturally…have an affinity to someone that looks like me, or behave like me… I think that's where the work needs to start, getting people really aware of their unconscious biases’. (Fam 01, 36 years, Arab, England)

#### 3.1.2 ‘What Defines Who I Am’

##### 3.1.2.1 Personal Circumstances

Participants did not report travel or socioeconomic barriers to attending appointments, even though almost two‐thirds of the participants were classed as socioeconomically deprived by IMD. Some chose to travel beyond their local district hospital to receive treatment based on positive experience of the care they received. Overall, participants expressed a willingness to go wherever necessary to receive the best treatment.

##### 3.1.2.2 Culture

Culture strongly shaped participants' experiences, influencing how they saw their role in the community and the challenges of being a woman living with breast cancer. One Arabian participant felt that culture rather than religion plays a more significant role in people's thoughts and actions. She acknowledged that moving to the United Kingdom had changed her cultural outlook.

Many participants across different ethnic groups perceived breast cancer as closely linked to stigma and felt that disclosing their diagnosis invited ‘gossip’, judgement or perceptions of failure, leading them to conceal their diagnosis (Quotes 1 and 2). Others avoided sharing their diagnosis due to intrusive questioning, which heightened anxiety and stress and may indicate limited awareness of cancer within the community (Quotes 3 and 4). Several participants realised after diagnosis that relatives had previously had cancer but not disclosed it. One participant described the relief of seeing other women ‘like me’ when she started chemotherapy, so felt less alone.

##### 3.1.2.3 Role in Society and the Family

Participants described navigating pre‐diagnosis challenges such as divorce, ill health and IVF treatment, which gave them strength and resilience. Some feared losing community status if their cancer became known, as it was suggested illness was often associated with weakness or pity (Quotes 1 and 2). Several participants highlighted the strong cultural expectations to provide emotional and practical support to family while continuing treatment and, in many cases, employment. Maintaining these roles gave several participants a sense of control, which was illustrated through talking about their priorities as a mother, wife and daughter during a time of uncertainty and feelings of perceived fatalism. Participants often prioritised their family's well‐being over their own (Quotes 3–6).

Putting their own needs aside created strain. One lady did not even discuss her cancer with her husband and led two separate lives: one as a mum and wife, the other as a patient (Quotes 7 and 8).

Some participants described the emotional impact on family relationships. Trying to meet cultural expectations while feeling unwell caused anguish and upset, and one participant described guilt of telling her children that she had cancer at a time when she felt at breaking point (Quotes 9–11). Others described strong support from close family and friends, which helped them to cope (Quotes 12–15). Many, however, described limited support from their wider family or community because of cultural views of cancer, geographical distance or older relatives' beliefs. There were nuances from participants that there were deep‐rooted cultural causes, indicating the struggles that some women can face following diagnosis. Minimal practical support from the health care system was also reported, often because services were hard to access, not conducive to their needs or not offered. Heavy family commitments left many participants with little capacity to seek information about treatment. Practical support that could alleviate some of the pressure, so that they were able to pursue the information they needed, was often reported (Quote 16).

##### 3.1.2.4 Religion and Faith

Religion and faith were central to all participant's psychological health and well‐being, shaping their beliefs and behaviours. The Quran was ‘a big anchor to hold on to’, and their religion and faith strengthened their resilience and acceptance of the disease, a feeling. echoed by family members (Quotes 1–3). Participants understood breast cancer through an Islamic worldview, describing illness as a test from God that could strengthen faith and spiritual reward (Quotes 4 and 5). At the same time, many emphasised that Islam encourages seeking medical treatment (Quote 6) and caring for the body as a responsibility (amanah) from God. Many referred to the hadith ‘Should I tie my camel or trust in Allah?’ The Prophet Muhammad replied: ‘Tie it and Trust in Allah’, to illustrate balancing personal responsibility with trust in God.

Religious coping provided hope, protection and inner peace, especially for women with secondary breast cancer (Quotes 7–9). Although all participants identified as Muslim, there was variation in how religious beliefs and practices were experienced and enacted. These differences reflected individual interpretation as well as personal, social and contextual circumstances. For example, one participant described praying primarily during times of need (Quote 10), while another reported that the emotional burden of cancer and competing responsibilities affected her ability to pray.

#### 3.1.3 Perception of Cancer

Beliefs about cancer influenced risk appraisal and decision‐making. Personal experiences of family and friends influenced whether they saw cancer as a treatable disease (Quotes 1 and 2).

##### 3.1.3.1 Influence of Cultural Beliefs

A key subtheme was the cultural meaning of cancer, which varied across and between ethnic groups and migration status. For some, cancer was often associated with fear, pain and suffering. One participant spoke of doctors and family members in Bangladesh not disclosing the diagnosis of breast cancer to women because they relate cancer to death (Quotes 1 and 2). Even when women knew survivors, their first thoughts were often of those who had died.

Cancer was widely described as a hidden condition associated with shame, taboo and embarrassment, particularly for women, making it difficult to discuss openly (Quotes 3–5).

Some participants felt breast cancer was not spoken about out of respect for modesty rather than it being taboo (Quote 6). Others described the emotional burden of feeling exposed or judged (Quote 7). Concerns about hereditary risk of cancer added further anxiety (Quote 8). Participants who were UK‐born or had lived in the United Kingdom for many years described greater openness and confidence discussing cancer, reflecting perceived cultural and generational differences (Quotes 9–11). These women also reported being more likely to seek information and support.

There was common recognition amongst participants that cancer treatments have improved, and participants felt they were receiving the best treatment, but treatment advances were not seen as connected to cancer research.

#### 3.1.4 Perception and Experience of Clinical Trials

##### 3.1.4.1 Awareness and Understanding

Awareness of clinical trials was limited, with many participants unfamiliar with the term, their purpose, processes and potential benefits (Quotes 1 and 2). Limited understanding often led to uncertainty and scepticism, including fear of being treated like a ‘guinea pig’ (Quote 3). Awareness and attitude towards research were shaped by intersecting factors such as migration history, language, education and cultural and religious beliefs, including the view that destiny is in the hands of God (Quotes 4–6).

Some participants linked awareness to education or profession, yet even those with higher levels of education reported difficulty understanding what participation would involve, and one did not realise that materials given related to research. This challenge was perceived to be greater for women whose first language was not English and/or who had limited formal education (Quotes 7 and 8). Family played a central role in decision‐making across cultures, with support from relatives increasing some participants' confidence when considering clinical trial participation (Quotes 9–11).

Participants from multiple ethnicities, who demonstrated understanding of clinical trials, recognised potential benefits, though some confused clinical trials with other types of research (Quotes 12–14). A few participants were aware of the underrepresentation of ethnic minorities in research and how this reinforced feelings of scepticism and mistrust across cultures (Quote 15). One Black African participant highlighted the need for more diverse trial participation after experiencing a side effect not previously documented. For some, especially those with advance disease, research offered hope and a chance to help others. Altruism, particularly towards other women in their communities, was a strong motivator (Quotes 16 and 17).

##### 3.1.4.2 Benefits vs. Risks

Participants' views on treatment‐related risks varied. Most described concerns about side effects of investigational treatments as a barrier to participation, while the prospect of therapies with fewer side effects was motivating (Quote 2). Two participants, who avoided chemotherapy through randomisation, viewed this positively, citing reduced toxicity and less impact on daily life (Quotes 3 and 4). Others were motivated if trials used therapies already used in practice, which they viewed as ‘safe and approved’. The findings suggested that personality traits could influence decisions, where participants spoke of some people as risk takers and more willing to participate despite uncertainties.

##### 3.1.4.3 Personal Experience of Clinical Trials

Irrespective of ethnicity and socioeconomic backgrounds, participants spoke of feeling frustrated and aggrieved due to failings in access to clinical trials. The findings indicated that participants' queries about clinical trials were sometimes dismissed by HCPs not involved in research, acting as gatekeepers and limiting access (Quote 1). One participant felt ‘cheated’ after learning another woman (non‐Muslim) had been offered a trial she had asked about, raising equity concerns. Family members reported that access to clinical trials often depends on HCPs' attitudes towards research. Experience of taking part in clinical trials varied, with findings signifying poor communication at enrolment and during the trial. One participant expressed anger at not being informed of trial blood results, used to monitor for treatment response and potential disease progression, which she perceived as unethical and highlighted a gap between trial protocols and women's expectations. Participants perceived close monitoring to be no different from standard care, with reports of feeling unsupported or abandoned (Quotes 2 and 3). Trials were described as demanding due to intensive follow‐up, physical and emotional strain and financial pressures.

### 3.2 Information and Education to Support Decision‐Making

#### 3.2.1 Information Source

Participants accessed breast cancer information from multiple sources: media stories, religious networks (Quote 1), printed materials and online resources. Peer discussions delivered through cancer charities were not always relatable (Quote 2). Initial hesitation about joining support groups was common, especially when women felt no one would be ‘like them’, but culturally concordant contact with an organiser helped build trust and engagement (Quote 3).

Social media was rarely used for medical guidance. Most participants preferred to rely on HCPs for information as online information could be overwhelming, even for those with high educational attainment (Quotes 4 and 5). Two participants relied heavily on medically trained family members for clarification and guidance during decision‐making, though it was suggested that many patients struggle when dependent on younger relatives to interpret complex information (Quotes 6 and 7).

Irrespective of language and literacy skills, participants emphasised the need for various information formats. Written information was often portrayed as unsuitable, even when translated, due to literacy barriers and low motivation to read them (Quotes 8 and 9). Verbal communication from trusted HCPs using clear, simple language was preferred. One participant with low education attainment still valued written materials for the ability to revisit information. A combined approach was also valued, simple written summaries alongside repeated verbal discussion to build trust and understanding (Quote 10). The findings suggest participants appreciated HCPs who share the same language and cultural background (Quote 11). Participants reported concerns with interpreters related to dialect, cultural sensitivity and reliance on the interpreter's understanding (Quote 12). Participants suggested training translators in basic clinical trial concepts to reduce miscommunication (Quote 13).

Short bilingual videos were widely supported, particularly in community settings, and recommended that cancer centres display brief informational videos in waiting areas. Although study‐specific videos were viewed positively, one participant highlighted that they may raise questions requiring further discussion (Quotes 14–16).

#### 3.2.2 Content and Clarity

Participants consistently wanted simple, clear, concise and culturally sensitive information to reduce fear and mistrust (Quotes 1 and 2). However, participants described variation in how research terminology is interpreted across communities. For example, ‘trial’ was associated with ‘trial and error’ by some participants, creating uncertainly, while ‘clinical’ was understood differently across Pakistani and Bangladeshi communities (Quotes 3 and 4). Regardless of educational attainment, participants preferred visual and engaging resources illustrating the cancer pathway, when clinical trials may be offered, and how previous research has improved treatments. They also requested greater transparency about eligibility, access to investigational treatments, and safety monitoring. Testimonials from women of similar cultural backgrounds were viewed as particularly valuable.

Study‐specific information, including trial benefits, risks, side effects, efficacy and participant demographics, was central to informed decision‐making, with one participant highlighting the importance of reporting toxicity across diverse ethnic groups (Quotes 5 and 6). Participants already enroled in trials also requested information on trial phases, similar studies and interim results to increase confidence in their treatment.

#### 3.2.3 Delivery

A recurring theme was the need for clinical trial information to be delivered in culturally appropriate ways. Community‐based women's groups were seen as the most effective setting, offering safe spaces where women could share experiences and receive information. Faith‐based venues were recognised as accessible for some, but participants highlighted that mosques are less frequently attended by women unless events are specifically organised (Quotes 1–3).

Participants also suggested culturally tailored social media, TV and radio campaigns, especially through specific community stations (Quote 4). A few felt education should begin in secondary schools, linking research to science lessons so younger generations can then help inform future generations, support relatives and reduce stigma, similar to one participant's experience in Nigeria (Quotes 5 and 6). Several participants highlighted the value of community champions who understand diverse cultural norms, appreciate language and literacy barriers and can bridge the gap between healthcare services and women who face socio‐cultural isolation or struggle to make decisions (Quote 7).

### 3.3 The Role of the HCP in Supporting Trial Participation

#### 3.3.1 Interpersonal Care

Participants strongly valued personalised holistic care from HCPs. Many described staff across all roles as ‘caring’, ‘helpful’ and ‘attentive’ and emphasised how hospital gardens, calm waiting areas and refreshments had a positive effect on physical and mental well‐being (Quotes 1–3). One participant felt regular personalised phone calls during treatment would be beneficial (Quote 4). Trusting relationships with HCPs, characterised by personalised and culturally sensitive care, were seen as essential. In their absence, participants described reduced motivation, with one participant questioning whether she would participate in a clinical trial again (Quotes 5–7).

#### 3.3.2 Building and Maintaining Trusting Relationships

The findings highlight that trust in HCPs strongly influenced decision‐making. Many participants relied on clinicians' expertise when feeling uncertain, and women enroled in clinical trials described trusting interpersonal relationships with research nurses. Some preferred doctors to guide treatment decisions, with one participant likening her trust in the medical profession to her trust in God (Quotes 1 and 2). Participants described differing expectations of decision‐making, with one family member highlighting diversity in cultural experiences; therefore, attempts by HCPs to promote individual autonomy around treatment choices could be perceived as uncomfortable if not handled sensitively (Quote 3).

Participants from across socioeconomic, language proficiency and cultural backgrounds spoke of feeling ‘ignored,’ ‘abandoned’ and let down by HCPs due to failings in care and access to support services (Quotes 4 and 5), including misdiagnosis and treatment delays leading to mistrust in the medical profession. Many were not offered support to raise concerns, despite staff acknowledging errors (quotes 6–8).

Perceived poor care experience quickly undermined confidence (Quote 9); one participant maintained she would ‘never step into’ her local Trust again (Quote 10) and some women linked mistrust to experiences in their native countries. One trial participant suspected doctors were financially incentivised to recruit patients (Quote 11), a view echoed by a family member who expressed broader scepticism about the medical profession and pharmaceutical companies.

#### 3.3.3 Cultural Sensitivity and Competency

Participants emphasised the importance of culturally competent care. They valued HCPs who understood religious beliefs relating to modesty, privacy and physical examination (Quote 1) and the acceptability of some medicines (Quote 2). However, participants also highlighted considerable cultural diversity within South Asian and Muslim communities, cautioning against assumptions based on ethnicity or religion alone (Quote 3).

Some participants perceived that unconscious bias contributes to missed opportunities to discuss clinical trials, particularly within busy clinical environments. A family member working in healthcare acknowledged that unconscious bias is universal, emphasising the need for awareness and mitigation. Participants recommended cultural‐competency training to equip HCPs to communicate effectively with patients from diverse cultural and religious backgrounds, support informed decision‐making and promote equitable access to clinical trials (Quotes 4 and 5).

## 4 Discussion

This study contributes to the sparse literature exploring Muslim women's experience of cancer; how faith, religion and cultural norms influence health beliefs and behaviours to participating in clinical trials. Fifteen Muslim women, including those whose first language is non‐English and representing diversity across ethnicity, age and socioeconomic deprivation, were interviewed. The following discussion maps the inductively derived themes onto the COM‐B model to represent a structured interpretation of the findings while preserving the epistemological integrity of RTA. In addition, the COM‐B framework can be embedded within the socio‐ecological model, which represents the dynamic interactions between the individual (intrapersonal), family, HCPs (interpersonal), community and societal [17] to provide a multilevel explanation of behaviour.

### 4.1 Capability

Participants' psychological capability of participating in cancer clinical trials was strongly influenced by knowledge, understanding and reasoning of cancer and clinical trials. Results reflect broader evidence that ethnically diverse communities' capability is constrained by inaccessible culturally relevant information [19, 46, 47]. Limited knowledge and understanding reinforce misconceptions and perpetuate mistrust and scepticism towards research, as others have highlighted [18, 19].

The results suggest women's awareness of cancer and clinical trials is influenced by diverse cultural and religious influences alongside intersecting social determinants, including migration history, generation, education, language and social position. For some participants, understanding of cancer was shaped by media coverage, family experience and cultural norms. Cancer was often hidden due to feelings of shame, embarrassment, concern over becoming subjects of social stigma, through informal community communication or fear of losing their status in society (self‐stigma). Social and self‐stigma are commonly reported to have negative outcomes, including limiting access to healthcare services and treatment [48, 49, 50, 51, 52]. Fear, shame, gossip and social judgement can limit opportunities to discuss cancer, reducing access to information and support. This in turn can reduce capability by reinforcing misunderstandings about cancer and clinical trials, while diminishing motivation to engage and seek information [16, 32]. Importantly, stigma varied by cultural background, generation and perceived acculturation, suggesting it reflects broader cultural and social contexts rather than religion.

Despite many participants reporting higher educational attainment, their accounts suggested difficulties interpreting research information, supporting evidence that education alone is not a reliable indicator of health literacy [24]. Women preferred verbal explanations, short videos or faith‐based communication over written materials, indicating that capability is enhanced when information is linguistically accessible, culturally meaningful and presented in formats that facilitate understanding [16, 19, 53, 54]. Participants also valued information delivered by people who ‘looked like them’, supporting evidence that culturally matched communication is beneficial [55]. Conversely, reliance on English language or predominantly digital resources may inadvertently reduce capability and broaden existing inequalities [56].

Linguistic barriers further constrained psychological capability. Having a diverse workforce, who are bilingual, to support the delivery of education and information about clinical trials to improve opportunities and capabilities of patients participating in clinical trials is well documented in the literature [47, 57]. Patient navigation models have improved clinical trial participation among ethnically diverse populations by providing emotional, practical and educational support [58, 59]. Consistent with these findings, RAs in this study facilitated culturally meaningful communication, supported participants' education and fostered trust throughout the research process. While involving HCPs from similar cultural backgrounds has been shown to facilitate recruitment of ethnically diverse participants [19, 47], findings from this study indicate that cultural responsiveness extends beyond shared ethnicity or religion. Participants valued HCPs who recognised the diversity of beliefs, cultural practices and individual circumstances within Muslim communities, alongside community‐based RA roles.

### 4.2 Opportunity

Women's opportunity to engage with clinical trials was shaped by interpersonal, organisational and community‐level factors. Family members created social opportunities by facilitating access to sources of informational, emotional, and practical support, particularly family members who were more educated, English‐speaking or familiar with the healthcare system. In these instances, family members acted as ‘decision partners’ [60], reflecting collective decision‐making norms in diverse communities [46, 61]. However, reliance on family could also restrict opportunities to access accurate information, as others have reported [19, 62].

Community engagement emerged as a key opportunity to raise awareness. Faith‐based settings, women's groups, and schools were seen as trusted environments for education and discussion. Although mosque‐based and faith‐informed interventions have demonstrated acceptability and effectiveness [63, 64], participants highlighted the value of HCPs collaborating with religious leaders, supporting educational sessions and providing opportunities for breast cancer survivors to share their experiences. However, caution should be taken when tailoring messages to avoid stereotypes given the diversity within Islam. A collaborative approach is necessary to identify appropriate tailored messages.

From a socio‐ecological perspective, community‐level interventions, including culturally tailored media campaigns, community champions and educational workshops, may enhance psychological capability by improving research literacy, increase social opportunity by delivering information through trusted community networks and strengthen motivation by normalising conversations about cancer and research while building trust between researchers and communities [65, 66, 67]. To support these approaches, bilingual RA roles could be embedded within NHS Research & Innovation departments, with funding incorporated into clinical trial costings to support equitable recruitment alongside existing community and cancer support services. Community needs assessments may further help researchers identify barriers to trial access and tailor engagement strategies [12, 68].

Equitable trial participation depends not only on patients' behaviour but also on HCPs and organisational behaviours that create opportunities for engagement. Our findings suggest that dismissive and discriminatory HCP behaviours can restrict social opportunity by acting as gatekeepers to trial awareness and recruitment, while reinforcing mistrust and reducing motivation to engage with research, consistent with previous research [13, 16]. These findings indicate that conscious and unconscious bias remain barriers to equitable trial access in the United Kingdom. However, opportunity can also be constrained by organisational factors, including limited HCPs' awareness of trials, insufficient training, time pressures and resource constraints [12, 16, 69]. Importantly, our findings challenge the perception that ethnically diverse communities are ‘hard to reach’ or unwilling to participate, suggesting that inequitable access reflects failures in opportunity rather than a lack of patient motivation. This supports evidence that women from ethnically diverse communities are equally willing to participate when offered the opportunity [14, 16, 18, 70]. Trusting interpersonal relationship is fundamental to supporting women's decision‐making and are evident across the study themes [19, 66]. Personalised communication, including proactive phone calls, continuity of care and time to address concerns, helped participants feel understood and respected. Such patient–HCP interactions can strengthen social opportunity through trust‐building, which can then enhance motivation to engage in clinical trial discussions. These findings mirror those of previous studies which looked at facilitators for engaging ethnic diverse patients into research [19, 66].

Evidence suggests that participation among ethnically diverse populations improves when research teams receive training in cross‐cultural communication [12, 71]. Therefore, the importance of HCPs undertaking culturally responsive practice is key. Cultural competency involves the ability to integrate cultural and linguistic understanding into healthcare delivery while recognising the diversity that exists within and across ethnic groups [58, 72].

### 4.3 Motivation

Women's motivation to participate in clinical trials was shaped by emotions and identity, including religion and altruism [15, 73]. Women with secondary breast cancer recognised they may not benefit directly but expressed solidarity with others and a desire to improve future outcomes. Hope of prolonged survival and potential cure also influenced decision‐making.

Our findings support previous research demonstrating that religion and culture influence health beliefs and behaviours among Muslim women [64] but show that these influences are neither uniform nor synonymous. While many participants viewed illness as part of God's divine plan, consistent with Islamic beliefs [64, 74], they also emphasised personal responsibility to seek treatment and care for the body entrusted to them by God. Faith therefore appeared to promote adaptive coping, acceptance of cancer and hope, suggesting that faith‐informed education may strengthen reflective motivation by aligning clinical trial participation with personal values [28, 75, 76].

Importantly, participants' health‐related decisions reflected the interaction of religion with culture, education, personal values and lived experience. Although modesty is a core Islamic principle [74], its influence on healthcare decisions varied considerably between women, reinforcing the need for HCPs to avoid assuming that religious affiliation predicts individual preferences. Instead, culturally responsive care should explore how each woman interprets and priorities her religious and cultural beliefs [77].

A recurring theme was women's competing priorities, which constrained both physical opportunity and reflective motivation to participate in clinical trials. Participants commonly prioritised family well‐being over their own, consistent with Islam et al. [52], reinforcing gendered caregiving expectations and limiting opportunities to engage in research. However, these roles were not universal; some participants described husbands and relatives assuming caregiving responsibilities during cancer treatment, illustrating how family support can mitigate practical barriers to participation. Nevertheless, participants acknowledged that traditional caregiving expectations continue to shape many women's cancer experiences. Previous research suggests that, in this context, research participation may be perceived as burdensome or even ‘selfish’, particularly when cancer already threatens family stability [78]. Family therefore represented an important source of social opportunity, acting as either facilitator or barrier to participation [19, 65]. These findings suggest that interventions should extend beyond individual patients by involving family members in clinical trial discussions to strengthen understanding, trust and shared decision‐making.

An important finding was that one participant explicitly stated that culture rather than religion is a more significant driver of her behaviour. This suggests that while religion may shape values and motivations, cultural and societal commitments and expectations may have a more direct influence on decision‐making and participation in clinical trials. These findings reinforce the importance of considering an individual's influencing ‘ecosystem’ and the concept of ‘intersectionality’ when attempting to engage communities in research [30]. The participants in this study shared other marginalised identities such as socioeconomic status, geographic background, and being female, which can increase vulnerability to unequal distribution of power that then intersects with health systems that are not sufficiently responsive to the needs of minoritised women [32]. Distinguishing between these influences is important as assuming religion alone determines healthcare behaviour risks reinforcing stereotypes and may overlook modifiable cultural or structural barriers to clinical trial participation [79].

## 5 Limitations

Study limitations include: (1) most participants resided in socioeconomically deprived areas, potentially limiting transferability, although this reflects the geographical distribution of many Muslim communities; (2) interview translation occasionally required summarising dialogue, reducing linguistic nuance and opportunities for probing; (3) formal participant validation was not undertaken, although ongoing involvement of the RA enhanced cultural interpretation; (4) despite bilingual transcript checking, back‐translation was not feasible for all interviews because of time and cost constraints; (5) the presence of a community‐based RA may have influenced disclosure of culturally sensitive experiences; and (6) self‐selection bias may have occurred, as participants may have held more favourable views towards research than non‐participants.

## 6 Conclusion

The study highlights the importance of recognising the diversity within Muslim communities and demonstrated that participation in cancer clinical trials is shaped by interacting behavioural, cultural and organisational influences rather than religion alone. Applying the COM‐B model showed that inequitable participation is driven by limited opportunities, including HCP gatekeeping, unconscious bias and inconsistent trial discussions, rather than a lack of willingness among ethnically diverse women. These findings support a shift from cultural competency towards culturally safe practice that encourages critical reflection on individual and organisational biases influencing equitable research access. Community‐based approaches, including partnerships with community organisations and RAs, offer a promising strategy to enhance capability, expand opportunity and strengthen motivation for clinical trial participation. Future research should evaluate the implementation and effectiveness of these approaches, including RA roles and culturally responsive training, to support more equitable participation across underserved communities.

## Author Contributions

**Lorraine Turner:** conceptualisation, investigation, funding acquisition, writing – original draft, methodology, validation, visualisation, writing – review and editing, formal analysis, project administration, data curation, software. **Sally Taylor:** methodology, validation, visualisation, writing – review and editing, formal analysis, supervision. **Ashleigh Ward:** writing – review and editing, formal analysis, project administration, validation. **Nighat Ahmed:** methodology, validation, writing – review and editing. **Janelle Yorke:** methodology, validation, writing – review and editing, supervision.

## Conflicts of Interest

The authors declare no conflicts of interest.

## Data Availability

The data that support the findings of this study are available on request from the corresponding author. The data are not publicly available due to privacy or ethical restrictions.
